# QTL mapping of rat blood pressure loci on RNO1 within a homologous region linked to human hypertension on HSA15

**DOI:** 10.1371/journal.pone.0221658

**Published:** 2019-08-23

**Authors:** Blair Mell, Xi Cheng, Bina Joe

**Affiliations:** Department of Physiology and Pharmacology, Program in Physiological Genomics, Center for Hypertension and Precision Medicine, University of Toledo College of Medicine and Life Sciences, Toledo, OH, United States of America; Medical College of Wisconsin, UNITED STATES

## Abstract

Fine-mapping of regions linked to the inheritance of hypertension is accomplished by genetic dissection of blood pressure quantitative trait loci (BP QTLs) in rats. The goal of the current study was to further fine-map two genomic regions on rat chromosome 1 with opposing blood pressure effects (BP QTL1b1 and BP QTL1b1a), the homologous region of which on human chromosome 15 harbors BP QTLs. Two new substrains were constructed and studied from the previously reported BP QTL1b1, one having significantly lower systolic BP by 17 mmHg than that of the salt-sensitive (S) rat (*P* = 0.007). The new limits of BP QTL1b1 were between 134.09 Mb and 135.40 Mb with a 43% improvement from the previous 2.31 Mb to the current 1.31 Mb interval containing 4 protein-coding genes (*Rgma*, *Chd2*, *Fam174b*, and *St8sia2*), 2 predicted miRNAs, and 4 lncRNAs. One new substrain was constructed and studied from the previously reported BPQTL1b1a having a significantly higher systolic BP by 22 mmHg (*P* = 0.006) than that of the S rat. The new limits of BPQTL1b1a were between 133.53 Mb and 134.52 Mb with a 32% improvement from the previous1.45 Mb to the current 990.21 Kb interval containing 1 protein-coding gene, *Mctp2*, and a lncRNA. The congenic segments of these two BP QTLs overlapped between 134.09 Mb and 134.52 Mb. No exonic variants were detected in any of the genes. These findings reiterate complexity of genetic regulation of BP within QTL regions, where elements beyond protein-coding sequences could be factors in controlling BP.

## Introduction

**Fig 1:**
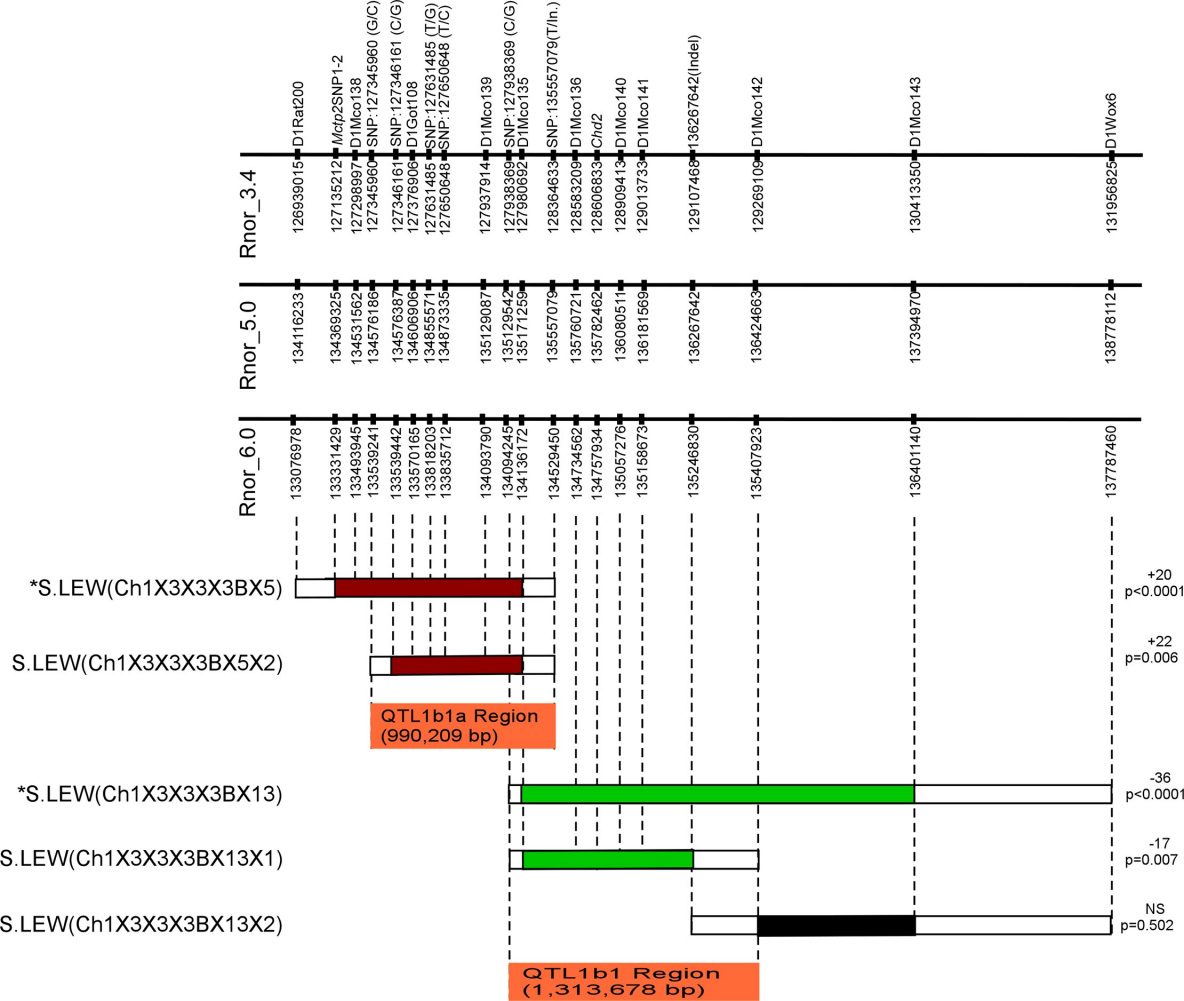
The schematic map of new congenic substrains. The physical map of RNO1 is shown along with the microsatellite and SNP markers with their locations according to the Ensembl database (www.ensembl.org, RGSC 3.4, 5.0 and 6.0). The horizontal bars below the physical map represent schematics of congenic strains. Introgressed LEW segments are shown with colored bars. A red bar indicates that the congenic strain had a significantly higher BP than S. A green bar indicates that the congenic strain had a significantly lower BP compared with S. A black bar indicates that the congenic strain had no significant change in BP compared with S. The white boxes flanking each of the congenic strains are regions of recombination. The BP effect of each strain compared with S is shown to the right of the schematic of each congenic strain. The locations of the newly identified BP QTLs are indicated as orange bars. *Data from S.LEW (Ch1x3x3x3Bx5) and S.LEW (Ch1x3x3x3Bx13) was previously published and is presented here for completeness [16].

Genetic analysis of quantitative traits in rat models has been shown to be a suitable approach to positional cloning of genetic determinants of polygenic traits [1–9]. One such complex polygenic trait is blood pressure (BP), which is extensively studied using rat models. Linkage of human hypertension to a large region on the q-terminus of human chromosome 15 has been detected by four independent studies [10–13]. This region in humans has been mapped to a location on rat chromosome 1 (RNO1) using homology mapping. BP quantitative trait loci (QTLs) in this region on RNO1 have been reported from previous linkage and substitution mapping studies [14–18]. Previously, two BP QTLs were located within 2.31 Mb and 1.45 Mb on rat chromosome 1, referred to as QTL1b1 and QTL1b1a respectively, using the congenic strains S. LEW (Ch1x3x3x3Bx13) and S.LEW (Ch1x3x3x3Bx5), respectively. These strains were developed by introgressing alleles from the normotensive Lewis (LEW) rat onto the genome of the hypertensive Dahl salt-sensitive (S) rat (Fig 1) [16]. These BP QTL regions were further resolved in the current study.

The importance of the current study is that both QTLs were further fine mapped into shorter segments. QTL1b1 was further fine mapped into two shorter segments: (1) S.LEW (Ch1x3x3x3Bx13x1) with <2 Mb, (2) S.LEW (Ch1x3x3x3Bx13x2) within 2 Mb (Fig 1). The first segment on QTL1b1 shows a decreased BP effect, whereas the second segment shows no BP effect. LEW alleles of QTL1b1 had a significant BP lowering effect of the S rat and were mapped within 1.314 Mb containing 10 annotations. QTL1b1a was further fine mapped into one shorter segment: S.LEW (Ch1x3x3x3Bx5x2) within <1 Mb (Fig 1). LEW alleles of QTL1b1a had a significant BP increasing effect of the S rat and were fine-mapped within 990 Kb containing 2 annotations. Genomic DNA sequencing data from these mapped locations has been cataloged. There were no exonic variants within the two newly defined QTL regions but several intronic and intergenic polymorphisms; therefore, these data point to variants outside protein-coding regions as potential candidates for the two BP QTLs with opposing BP effects.

## Methods

### Animals and diet

All animal research protocols described in this study were approved by the University of Toledo’s Institutional Animal Care and Use Committee. Experiments were performed in accordance with the National Institutes of Health Guide for the Care and Use of Laboratory Animals. The inbred Dahl salt-sensitive (SS/Jr or S) rat strain was maintained at The University of Toledo College of Medicine and Life Sciences. The Lewis (LEW) rats, originally obtained from Charles River Laboratories (Wilmington, MA), were maintained in our animal facility. In brief, the F1 population was generated by crossing the parental congenic strain with S. The F2 population was generated by intercrossing the F1 rats. The F2 population was genotyped using microsatellite markers throughout the region on rat chromosome 1 from marker D1Rat200 (Chr1:133076978) to D1Wox6 (Chr1:137787460, Rnor 6.0) (Fig 1). The recombinant F2 rats with various introgressed regions of the LEW alleles were backcrossed to S, sorted by genotyping, and then intercrossed to generate homozygous congenic animals on the genomic background of the S rat. S.LEW (Ch1x3x3x3Bx5) is the parental strain for the congenic substrain S.LEW (Ch1x3x3x3Bx5x2). S.LEW (Ch1x3x3x3Bx13) is the parental strain for the congenic substrains S.LEW (Ch1x3x3x3Bx13x1) and S.LEW (Ch1x3x3x3Bx13x2). Rats were maintained on a low salt diet (0.3% NaCl; Harlan Teklad diet 7034, Madison, WI). For experiments involving a high salt regimen (2% NaCl), the Harlan Teklad diet (TD94217) was used.

### Microsatellite markers

New microsatellite markers were developed from sequences downloaded from rat genome sequence data available (RGSC 3.4 or RGSC 5.0) through the Ensembl database (http://www.ensemble.org). Marker names and details are deposited with the Rat Genome Database (http://rgd.mcw.edu/). Sequence information on the new polymorphic markers with the prefix D1Mco is provided in the S1 Table. Primer 3 software (http://bioinfo.ut.ee/primer3-0.4.0/primer3/) was used to design primers around dinucleotide or trinucleotide repeats. Primer pairs were used to test for polymorphisms between S and LEW genomic DNA. The primer pairs that were polymorphic were used for genotyping F2, backcross, and intercross animals along with DNA from S and LEW as controls.

### Genotyping

Congenic substrain DNA was extracted from a tail biopsy using the Promega Wizard SV 96 Genomic DNA Purification System (Promega, Madison, WI, USA). PCR-based genotyping using microsatellite markers was performed using standard techniques as described previously [17].

### Blood pressure measurements by radiotelemetry

Rats were weaned at 28–30 days of age and maintained on a low-salt diet (0.3% NaCl). In order to match the blood pressure QTL inference drawn from the previous study of male rats, only male rats were used for the current study [16]. For each congenic strain, about 12–14 male rats were matched by age and weight to an equal number of male S rats. Each cage contained one congenic strain rat and one S rat to minimize environmental effects. At 40–42 days of age, rats were switched to a high-salt diet (2% NaCl) for an additional period of 24 days. After 24 days on high-salt diet, rats were surgically implanted with HD-S10 (S vs. Ch1x3x3x3Bx5x2) or C40 (S vs. Ch1x3x3x3Bx13x1) radiotelemetry transmitters (Data Science International, St Paul, MN) as previously described [14]. Rats were individually housed and allowed to recover from surgery for 3 days before recording blood pressure.

### Quantitative real-time PCR

Heart, kidney, brain, and spleen samples were collected from 10 week old S and S.LEW (Ch1x3x3x3Bx13x1) rats that were fed a high-salt (2%) diet. RNA was isolated using the TRIzol method following the recommended procedures (TRIzol, Invitrogen). Concentration of RNA was determined using Nanodrop 2000C. Total RNA was reversed transcribed to cDNA using Superscript III kit (Invitrogen). Then the cDNA was diluted and used as a template for quantitative PCR using SYBR Green. Levels of mRNA expression of *Chd2* were analyzed by real-time PCR on a Step One Plus® Real Time PCR Detection System (Applied Biosystems, Foster City, CA), as described previously [15]. By NCBI BLAST of the rat genome, primers were selected for specificity. Using the Step One Plus® Real Time PCR Detection System software, first derivative melting curve analysis verified amplicon specificity. Quantification and normalization of relative gene expression was accomplished using the comparative threshold cycle (C_T_) method or ΔΔ C_T_. ΔΔ C_T_ values were converted into ratios by 2^- ΔΔ CT^ and averaged across biological replicates. The expression of the “housekeeping” gene ribosomal protein L36a (GenBank accession no. AA859783) was used for normalization, as this gene did not exhibit differential expression as previously shown [15].

### Genomic sequencing and analysis

Genomic variants between SS/Jr and LEW/Crl were obtained from the Rat Genome Database (http://rgd.mcw.edu/). Primers were synthesized by Integrated DNA Technologies (Coralville, IA) and used for manual sequencing. PCR products were sequenced through the Eurofins MWG Operon (Huntsville, AL) sequencing service and sequencing data was analyzed using Sequencher 4.10.1.

### Statistical analysis

All statistical analyses of blood pressure studies were conducted using GraphPad Prism 5 (version 5.02). Data was analyzed by independent sample Student’s *t*-test. The data is presented as the mean ± standard error (Mean ± SEM). A p-value of <0.05 was used as a threshold for statistical significance.

## Results

**Fig 2:**
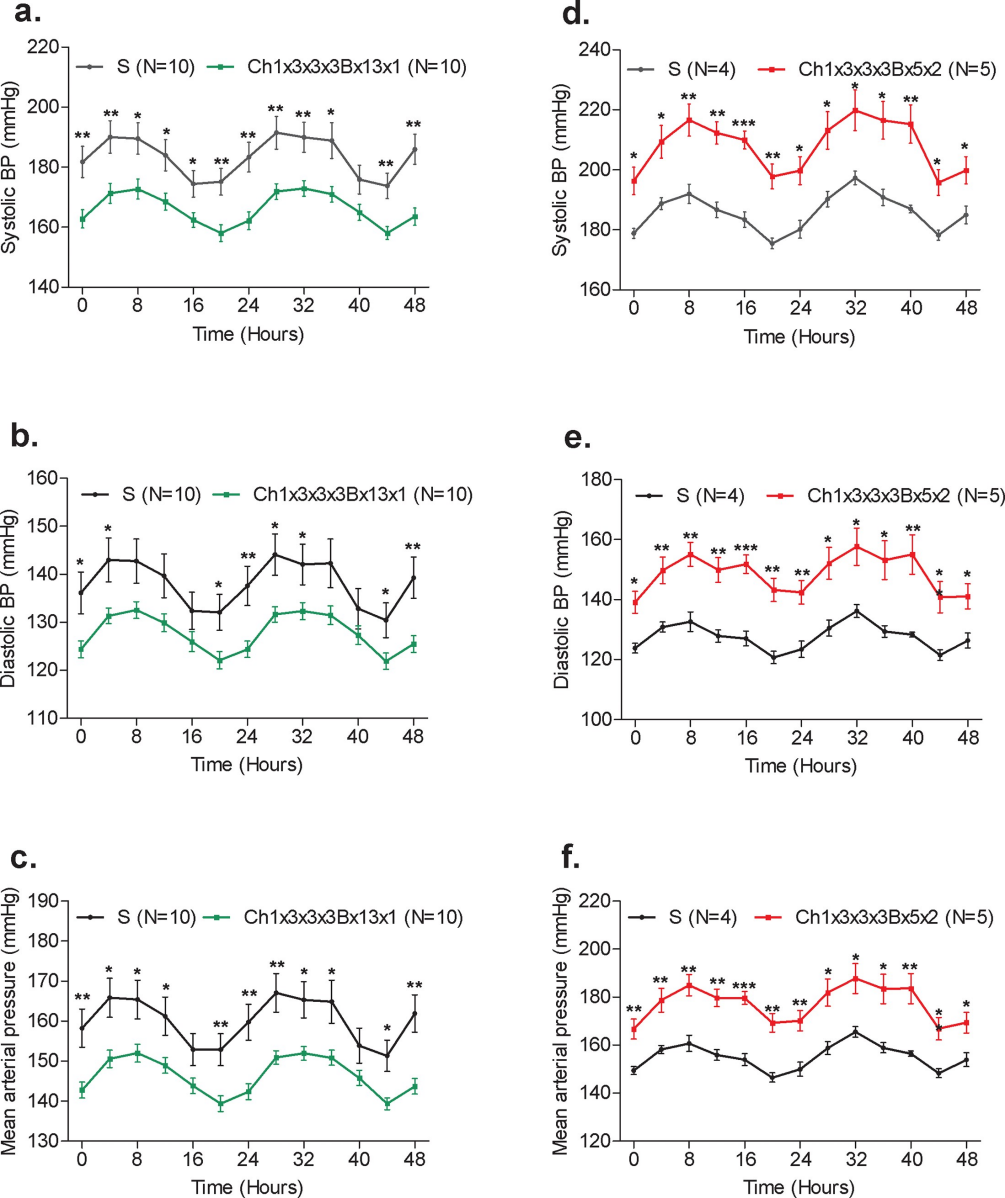
Radiotelemetry blood pressure measurements. Radio-transmitters were implanted into S and congenic strains and their BP was continuously recorded for 48 hours as detailed under the Methods. n = 4–10 rats/group. Systolic BP, systolic blood pressure (a,d); Diastolic BP, diastolic blood pressure (b,e); Mean arterial pressure (c,f). *A p-value of <0.05; **A p-value of <0.01; ***A p-value of <0.001.

**Fig 3:**
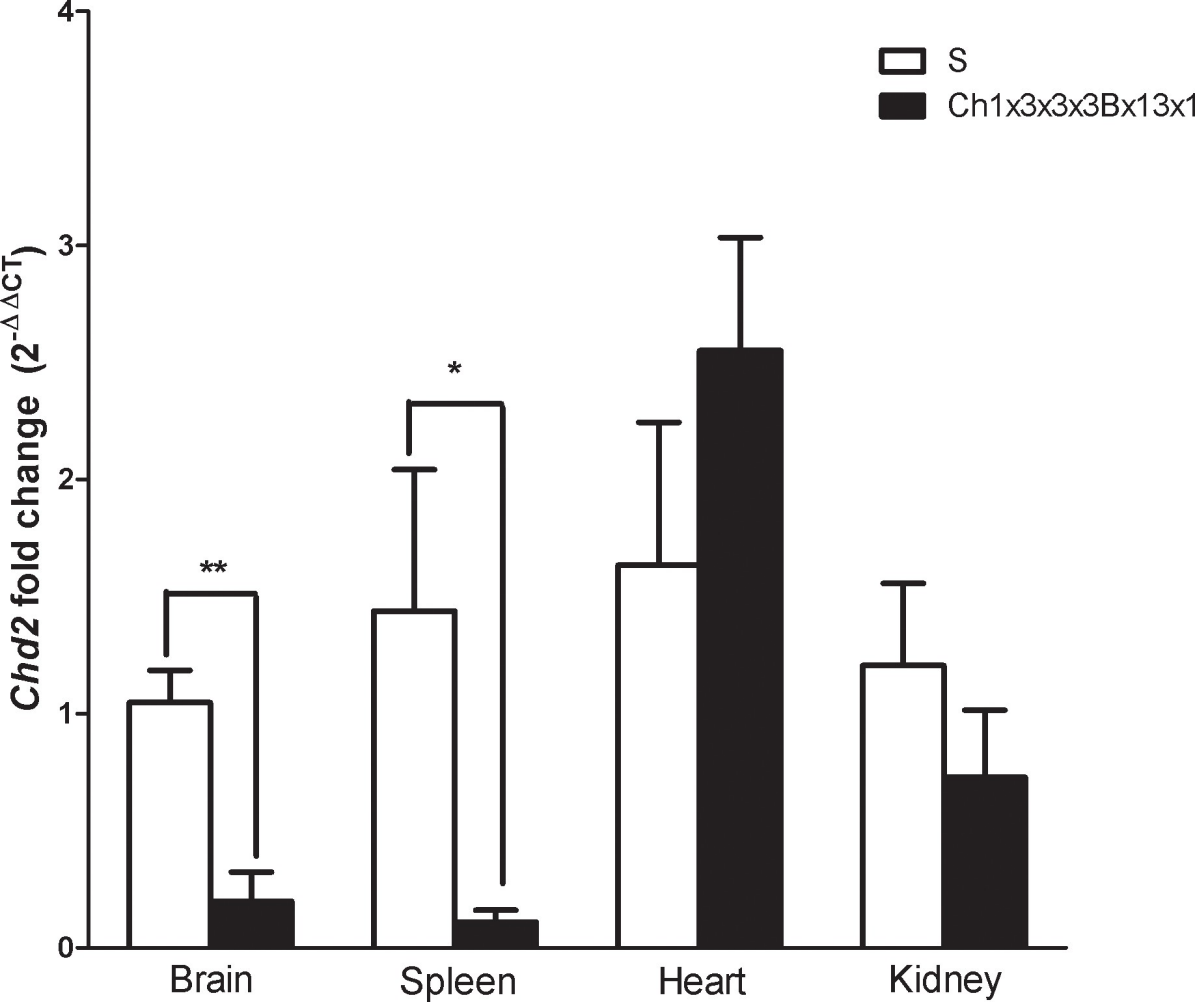
Transcript expression analysis of Chd2 in S and S.LEW (Ch1x3x3x3Bx13x1). Real-time PCR data from S and S.LEW (Ch1x3x3x3Bx13x1) congenic substrain. mRNA obtained from brain, spleen, heart and kidney samples were converted to cDNA then amplified using specific primers. Values are fold change ±s.e.m. **P≤0.01, *P≤0.05.

The previously reported congenic substrain S.LEW (Ch1x3x3x3Bx13) had a significant BP lowering effect of -36 mmHg compared with the S rat and spanned the entire 2.31Mb QTL1b1 region from 134.09 to 136.40 Mb [16]. QTL1b1 has been further fine-mapped by constructing and characterizing 2 new congenic substrains S.LEW (Ch1x3x3x3Bx13x1) and S.LEW (Ch1x3x3x3Bx13x2) (Fig 1). These congenic substrains were developed by introgressing LEW rat alleles into the genome of the S rat. One of the newly constructed congenic substrains, S.LEW (Ch1x3x3x3Bx13x1) had a significantly lower systolic BP of 173 ± 7 mmHg compared to the systolic BP of the S rat, 190 ± 4 mmHg (*P* = 0.007). The systolic, diastolic and mean BP of the congenic strain S.LEW (Ch1x3x3x3Bx13x1) were lower than that of the S rat observed using the telemetry method (Fig 2). The other newly constructed congenic substrain, S.LEW (Ch1x3x3x3Bx13x2), showed no significant difference in BP (190 ± 2 mmHg) compared to the S rat (192 ± 3 mmHg; *P* = 0.502). Therefore the location of QTL1b1 is further fine-mapped within the congenic segment of S.LEW (Ch1x3x3x3Bx13x1) (Fig 1). The new QTL1b1 location on RNO1 was within a 1.31Mb region from 134.09 to 135.40 Mb. This shows a 43% improvement from the 2.31Mb region to the new 1.31Mb region of QTL1b1. The new 1.31Mb QTL1b1 region contains 4 protein-coding genes (*Rgma*, *Chd2*, *Fam174b*, and *St8ia2*), 2 predicted miRNAs, and 4 lncRNAs (Table 1). *Chd2* (Chromodomain helicase DNA binding protein 2) was prioritized as a candidate gene based on the functional implication that it modifies chromatin structure. Quantitative real-time PCR on heart, kidney, spleen, and brain tissue was performed to determine whether the mRNA expression levels of *Chd2* differed between the congenic substrain S.LEW (Ch1x3x3x3Bx13x1) and the S rat. The results indicated a significant down-regulation of *Chd2* in brain and spleen in the S.LEW (Ch1x3x3x3Bx13x1) strain compared with S (*P* = 0.001, *P* = 0.037, respectively) (Fig 3). No significant difference in expression level was seen in the hearts or kidneys (*P* = 0.273, *P* = 0.323, respectively).

**Table 1:** Annotations within QTL1b1.

Symbol	Name	Location (Rnor 6.0)	Homologous regions in Human
ENSRNOG00000048799	Novel miRNA	134,605,321–134,605,443	NA
*Rgma*	repulsive guidance molecule family member A	134,699,299–134,742,514	Chr 15: 93,042,868–93,088,198
*Chd2*	chromodomain helicase DNA binding protein 2	134,757,934–134,871,167	Chr 15: 92,905,067–93,022,512
LOC108349594	Known lincRNA	134,873,400–134,887,841	Chr 15: 92,884,041–92,898,482
ENSRNOG00000051226	Known lincRNA	134,921,673–134,922,407	Chr 15: 92,836,547–92,837,281
ENSRNOG00000041447	Novel miRNA	134,946,880–134,946,944	NA
ENSRNOG00000051426	Novel lincRNA	134,951,356–134,951,760	Chr 15: 92,813,676–92,814,080
*Fam174b*	family with sequence similarity 174, member B	135,083,974–135,162,685	Chr 15: 92,580,602–92,659,313
ENSRNOG00000051304	Known lincRNA	135,135,833–135,162,693	Chr 15: 92,593,990–92,620,850
*St8sia2*	ST8 alpha-N-acetyl-neuraminide alpha-2,8-sialyltransferase 2	135,336,374–135,406,700	Chr 15: 92,356,975–92,427,301

The previously reported congenic substrain S.LEW (Ch1x3x3x3Bx5) had a significant BP increasing effect of +20 mmHg compared with the S rat and spanned the entire 1.45Mb QTL1b1a region from 133.08 to 134.53 Mb [16]. QTL1b1a was further fine mapped by constructing and characterizing 1 new congenic substrain, S.LEW (Ch1x3x3x3Bx5x2) (Fig 1). S.LEW (Ch1x3x3x3Bx5x2) had a significantly higher systolic BP of 209 ± 5 mmHg compared to the systolic BP of the S rat, 187 ± 3 mmHg (*P* = 0.006) (Fig 2). Systolic, diastolic, and mean BPs of S.LEW (Ch1x3x3x3Bx5x2) were higher than that of the S rat observed using the telemetry method (Fig 2). Therefore the location of QTL1b1a has been further fine-mapped within the congenic segment of S.LEW (Ch1x3x3x3Bx5x2) (Fig 1). The newly mapped location of QTL1b1a on RNO1 was within a 990Kb region to which QTL1b1a was previously mapped. This represents a 32% improvement from the 1.45Mb region to which QTL1b1a was previously mapped. The newly mapped 990Kb QTL1b1a region contains 1 protein-coding gene, *Mctp2*, and 1 annotated lncRNA (Table 2). While there were several intronic and intergenic polymorphisms within this region, there were no exonic variants. The exonic variants of *Mctp2* lie outside of the QTL region. Gene expression of *Mctp2* was previously studied and found to have no significant difference between the previously reported QTL1b1a congenic strain and the S rat [16]. The congenic segments of these two new QTLs overlapped between 134.09Mb and 134.52Mb (Fig 1). The majority of the variants in both QTL regions were located within either intergenic or intronic regions (Table 3). The variants located in highly conserved regions were located within intergenic regions of QTL1b1a. Locations, SNPs and conservation scores are provided in the S2 Table. Genomic variant data within the two QTL regions between SS/Jr and LEW/Crl along with conservation scores were obtained from the Rat Genome Database (http://rgd.mcw.edu/).

**Table 2:** Annotations within QTL1b1a.

Symbol	Name	Location (Rnor 6.0)	Homologous regions in Human
*Mctp2*	Multiple C2 domains transmembrane 2	133,324,301–133,559,975	Chr 15: 94,231,538–94,483,952
ENSRNOG00000053587	Novel lincRNA	134,028,907–134,030,448	Chr 15: 93,813,356–93,814,897

**Table 3:** Regions of single-nucleotide polymorphisms between SS/Jr and LEW.

Location	Number of exonic variations (protein coding gene)	Number of intronic variations (#-Gene)	Number of intergenic SNPs (# of SNPs in highly conserved regions)	Number of 3’ and 5’ UTR SNPs (Gene)
QTL1b1a	0	• 36-*Mctp2* • 1-ENSRNOG00000053587 • 27-pseudogenes	859 (20)	36 (*Mctp2*)
QTL1b1	0	1 -*Fam174b*	24	0

**Fig 4:**
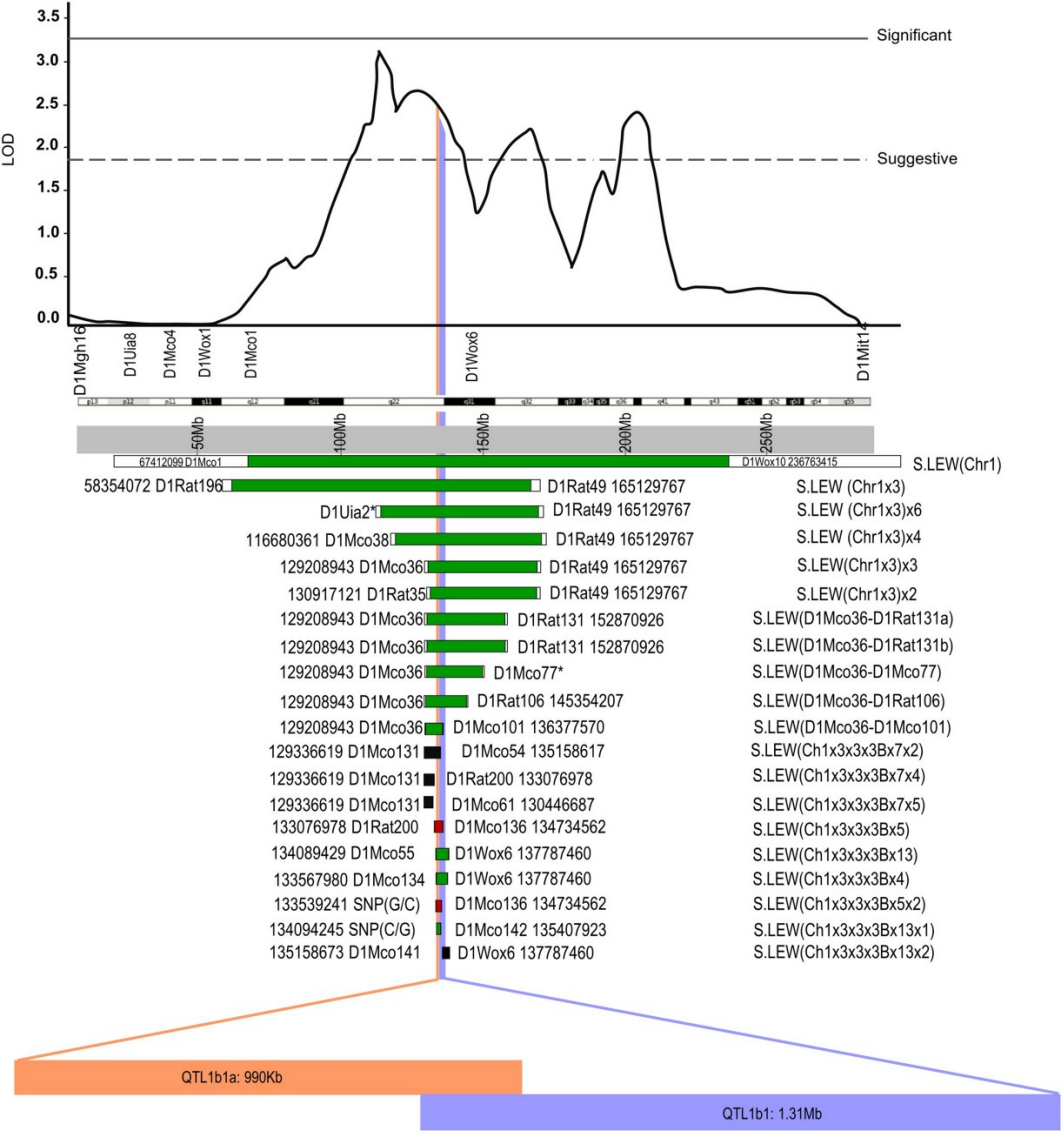
Linkage and substitution mapping on RNO1 using S and LEW rats. The LOD plot for BP was obtained from the study of an F_2_(S x LEW) population. This is shown at the top followed by the cytogenetic and physical maps of RNO1 [19]. End markers of congenic strains and current physical locations are shown with flanking bars. The physical locations of markers were obtained from the Rat Genome Database (http://www.rgd.mcw.edu) version 6.0. *The location for markers not mapped were determined using nearby markers chosen using the limits of the congenic strains. Congenic strains having a BP lowering effect are shown with a green bar. Congenic strains having no BP effect are shown with a black bar and a red bar indicates that the congenic strain has a BP increasing effect. All congenic strains except the ones currently reported are previously published [14, 16–19]. Vertical blue or orange lines collectively depict the reported QTL regions. The names of QTLs are depicted in the blue or orange horizontal bars along with their respective sizes.

Fig 4 illustrates these two independently functional BP QTLs within the originally detected logarithm of odds (LOD) plot for chromosome 1 by substituting LEW alleles into the S rat genome. QTL1b1 and QTL1b1a are now resolved to regions with candidate variants residing entirely outside the exonic regions of both QTL regions.

## Discussion

While genetic dissection of the human genome is difficult, substitution mapping in rats allows for further dissection of BP QTLs which can be comparatively mapped with human hypertension loci. The subject of this study are two such BP QTLs in the rat which are comparatively mapped within a large region encompassing the q-terminus of human hypertension loci on chromosome 15 [20–22]. This report is a new iteration of substitution mapping of LEW alleles on the S genome on RNO1, wherein two BP QTL regions were previously located within a 2.31Mb and 1.45Mb interval. Data collected from three new congenic substrains showed further mapping of the 2.31Mb BP QTL to a shorter 1.3Mb BP QTL with a BP lowering effect and the 1.45Mb BP QTL to a shorter 990Kb BP QTL with a BP increasing effect. Closely linked BP loci with opposing effects have been shown on other regions of the rat genome [23–25]. This phenomenon shows the complex genetic landscape on BP control in both rats and perhaps in humans.

Further comprehensive mapping of the previously reported 2.31Mb and 1.45Mb intervals has been shown in the current study by three new congenic strains. Genome sequencing of the QTL regions detected a large number of intronic and intergenic sequence variants but no exonic variants. The mapping and sequencing data taken together point to variants beyond protein coding sequences contributing to the BP effects of the two newly mapped genomic intervals on chromosome 1. This data is not surprising because large numbers of intergenic variants associated with several polygenic traits have been cataloged using genome-wide association studies (http://www.genome.gov/gwastudies/). Designing experiments to test the functional relevance of these variants to mechanisms underlying BP control is very difficult when the variants lie outside of protein coding regions. It is possible that these regions encompassing variants within introns and intergenic regions may interact with other chromosomal segments through chromatin remodeling. Therefore, we have prioritized *Chd2*, a gene located within QTL1b1, as a candidate gene influencing blood pressure. *Chd2* has been shown to regulate chromatin and is necessary for appropriate gene expression upon differentiation in mouse embryonic stem cells [26]. Enhancers have been shown to play a role in gene regulation and can regulate a gene from over a million base pairs or more in mammals [27]. Live imaging can be used to learn how enhancers are able to turn a gene on and off over long range distances [28]. Circular chromosome conformation capture (4C) sequencing has been used in recent studies to identify candidate genes for diseases such as atherosclerosis and chronic kidney disease through chromatin interactions with DNA regulatory elements [29, 30]. Therefore, we propose that chromosome conformation capture assay may be an important next step to further prioritize *Chd2* as a candidate gene for BP regulation via its effect on the folding of the genome.

## Supporting information

S1 Table Newly identified polymorphic markers. (DOC)

S2 Table Single-nucleotide polymorphisms between SS/Jr and LEW in highly conserved locations. (DOCX)
