# Supplementary material for: QTL mapping of rat blood pressure loci on RNO1 within a homologous region linked to human hypertension on HSA15
Source: PLoS One. 2019 Aug 23;14(8):e0221658. doi: 10.1371/journal.pone.0221658 (PMC6707578; doi:10.1371/journal.pone.0221658)
Supplement: S1 Table — (DOC) [file pone.0221658.s001.doc]

**Supporting Material**

**S1 table. Newly identified polymorphic markers**

| **Marker name** | **Forward** | **Reverse** | **Location in**  **Rnor 6.0** | **Location in**  **Rnor 5.0** | **Location in**  **Rnor 3.4** | **Size** |
| --- | --- | --- | --- | --- | --- | --- |
|  |  |  |  |  |  |  |
| D1Mco138 | CCTCAGAGGACCAAGTGGAA | CACACATTTGTGGGAACCAG | 133493945- 133494191 | 134531562- 134531808 | 127298997- 127299243 | 247 |
| D1Mco139 | TTCCTGGTAGCGAAGAATAGAG | TGGTTTTTATTTCACTTTGGTCTG | 134093790- 134094027 | 135129087- 135129324 | 127937914- 127938151 | 238 |
| D1Mco140 | TCATCATGGCAGGAGCATTA | TCTGTTCACCTCTGGCTGTG | 135057276- 135057524 | 136080511- 136080759 | 128909413- 128909661 | 249 |
| D1Mco141 | AATGGCTGCTTCTAATGTTCA | GGGGAAGTGGGTTCAAACTA | 135158673- 135158919 | 136181569- 136181815 | 129013733- 129013977 | 245 |
| D1Mco142 | TTCCCTGCTTCAGATGCTTT | GGAACCTTTCATCAGCCAAA | 135407728- 135407923 | 136424468- 136424663 | 129269109- 129269304 | 196 |
| D1Mco143 | ATTTGCCACTGCAGGACTCT | AGAAATCTGCCACTTTCAGACA | 136401140- 136401368 | 137394970- 137395198 | 130413350- 130413578 | 229 |
