# Supplementary material for: QTL mapping of rat blood pressure loci on RNO1 within a homologous region linked to human hypertension on HSA15
Source: PLoS One. 2019 Aug 23;14(8):e0221658. doi: 10.1371/journal.pone.0221658 (PMC6707578; doi:10.1371/journal.pone.0221658)
Supplement: S2 Table — (DOCX) [file pone.0221658.s002.docx]

**S2 Table. Single-nucleotide polymorphisms between SS/Jr and LEW in highly conserved locations**

| Location | Brown Norway | LEW | SS/Jr | Conservation Score^a^ |
| --- | --- | --- | --- | --- |
| 133561099 | G | G | C | 1 |
| 133574389 | T | T | C | .978 |
| 133638534 | C | C | T | .993 |
| 133683655 | C | C | T | .814 |
| 133698737 | G | G | A | .757 |
| 133730960 | C | C | T | .82 |
| 133737382 | C | C | A | .897 |
| 133776660 | A | A | T | .825 |
| 133829274 | G | G | A | .996 |
| 133831857 | C | C | T | .939 |
| 133831910 | C | C | A | .988 |
| 133865297 | G | G | A | .956 |
| 133873960 | A | A | G | .882 |
| 133913477 | A | A | T | 1 |
| 133913478 | C | C | T | 1 |
| 133958690 | A | A | G | 1 |
| 133958748 | T | T | C | 1 |
| 133958875 | G | G | A | .991 |
| 134075682 | C | C | T | .992 |
| 134093263 | T | T | G | .995 |

^a^ Conservation scores of 0.75-1 are highly conserved regions from Rat Genome Database
